# Supplementary material for: Sex and species specific hearing mechanisms in mosquito flagellar ears
Source: Nat Commun. 2018 Sep 25;9:3911. doi: 10.1038/s41467-018-06388-7 (PMC6156513; doi:10.1038/s41467-018-06388-7)
Supplement: Supplementary file 1 — Supplementary Information [file 41467_2018_6388_MOESM1_ESM.pdf]

Supplementary information for

Sex and Species Specific Hearing Mechanisms in Mosquito Flagellar Ears

Matthew P. Su[1,2,3], Marta Andrés[1,3], Nicholas Boyd-Gibbins[1], Jason Somers[1,3] & Joerg T. Albert[1,2,3,4]*

Affiliations:

[1]Ear Institute, University College London, 332 Gray's Inn Road, London WC1X 8EE, UK.

[2]Centre for Mathematics and Physics in the Life Sciences and Experimental Biology (CoMPLEX), University College London, Gower Street, London WC1E 6BT, UK.

[3]The Francis Crick Institute, 1 Midland Road, London NW1 1AT, UK.

[4]Department of Cell and Developmental Biology, University College London, Gower Street, London WC1E 6DE, UK.

*Correspondence to: joerg.albert@ucl.ac.uk

Supplementary methods

Power gain calculations: In order to cross-calibrate our newly devised procedure to calculate power gains with the methodology previously published[1], we applied it to *Drosophila* wildtype controls and compared the measured power gains resulting from our new approach to the power gains predicted from the previous model (which was made for an antennal receiver population of ~5ng apparent mass[1]). The ratios of measured and predicted power gains showed a strong correlation with the respective apparent mass of the system (determined as described above), with ratios of one (i.e. identical values for measured and predicted power gains) occurring for antennal mass values of 5ng. This demonstrates that for an identical population of receivers, our new approach produces identical energy estimates as precursor approaches (Supplementary Figure 2e). It has the benefit that by comparing the same receiver in two states (active/passive) it does not require any knowledge of further intrinsic system parameters, such as the receivers' apparent mass.

Sex-specific protocols for free fluctuation fitting: The best frequencies of the active and passive systems of female *Ae. aegypti* and *Cx. quinquefasciatus* mosquitoes are highly similar, with less than 10Hz difference calculated between them (Table 1). Thus, fitting the forced damped harmonic oscillator function to the active state data required no adjustments to be made for the passive system.

For female *An. gambiae*, the best frequency of the passive system was approximately 100Hz greater than that of the active system; this is similar to that reported for *Drosophila melanogaster* (though the frequency difference for *Drosophila* is far greater than calculated here for *An. gambiae* females); thus similar procedures to those used for *Drosophila* can be applied. Supplementary Figure 1a (left) contains examples of the fits for active and passive states for an *An. gambiae* female mosquito.

For male mosquitoes from each species, the best frequency of the passive system was estimated at around 200Hz less than that of the active system. Thus the free fluctuations of male mosquitoes in the active state show two peaks – one at around 300Hz representing the passive state and one somewhere between 450-550Hz representing the active state (as this peak disappears during sedation but returns once the mosquito has recovered). Therefore, in order to evaluate the active state, the damped harmonic oscillator function was fit only to the second peak (Supplementary Figure 1a right). Thus for low frequencies (around the best frequency of the passive state) the fit underestimates the energy of the active system as compared to the passive system; from the raw data it is clear that the free fluctuations recording are identical around these frequency ranges in both active and passive systems (see Supplementary Figure 1a right; Supplementary Figure 1b).

Effect of WN stimulus intensity on flagellar best frequency: During the white noise stimulus experiments, fluctuations of the flagellum were recorded for each stimulus intensity. This allowed for fitting the damped harmonic oscillator function to the data at each intensity, thus enabling estimation of the flagellar best frequency. The ratio of these best frequencies and the best frequency of the unstimulated flagellum was then calculated at each frequency. These values were amalgamated across different groups to allow for estimation of medians and standard errors (Supplementary Figure 1d).

Mosquito flagellar length measurements: Male and female mosquitoes were first sedated on ice and then held by the thorax in a pair of inverted forceps. Using a pair of sharpened forceps under a microscope, the flagella of each mosquito were removed from the pedicels and placed into a buffer solution. The flagella were then transferred to a microscope slide and a LSM 800 Zeiss confocal microscope (Zeiss, Cambridge) was used to examine each individual flagellum. Images were taken of intact flagella. These images were analysed using the LSM image browser software, which enabled measurement of the length and width of each flagellar section.

Radial symmetry of the mosquito flagellum was assumed, meaning that the maximum length and width of each flagellomere was assumed identical in every 2-dimensional plane.

8 *Ae. aegypti* female flagella, 10 *Ae. aegypti* male flagella, 23 *Cx. quinquefasciatus* female flagella, 16 *Cx. quinquefasciatus* male flagella, 9 *An. gambiae* female flagella and 22 *An. gambiae* male flagella were included in the final analysis. This allowed for estimation of the median total length of the flagellum as well as the distance between the point on the flagellum at which the laser was focussed and the base of the flagellum (Supplementary Table 1).

Converting flagellar displacements into angular deflections: Data analysis of the force step stimulation recordings was completed as described in the Methods section entitled ‘Force step stimulation recordings’. Flagellar displacements were then translated from nanometres to radians using the formula:

$$\text{Angular displacement } [rad] = \frac{\pi}{180} * \sin^{-1}\left(\frac{\text{displacement } [m]}{\text{calculated flagellar length } [m]}\right)$$

Supplementary Eq. 1

The torque at each displacement was then calculated using:

$$\text{Torque } [Nmrad^{-1}] = \frac{\text{Effective force } [N] * \text{calculated flagellar length } [m]}{\text{Angular displacement } [rad]}$$

Supplementary Eq. 2

The slope stiffness of the flagella at each displacement was then calculated from these values. Supplementary Figure 4 contains slope stiffness and antennal nerve response magnitudes for angular deflections of both female and male *Ae. aegypti*, *Cx. quinquefasciatus* and *An. gambiae* flagella.

Force step stimulation recordings with pymetrozine injection: Mosquitoes were mounted and exposed to force step stimulation as described in the Methods section entitled 'Force step stimulation recordings'. A ringer control solution was then injected into the mosquitoes and the force step procedure was repeated. Following this, a pymetrozine solution (at the same concentration as described above) was injected into the mosquitoes, after which the force step procedure was again repeated. Analysis of this data proceeded as described in the Materials and Methods section entitled 'Force step stimulation recordings'. Supplementary Figure 3 contains slope stiffness and antennal nerve response magnitudes for flagellar displacements of female and male *Ae. aegypti, Cx. quinquefasciatus* and *An. gambiae* both before and after pymetrozine injection.

Supplementary Figure 1

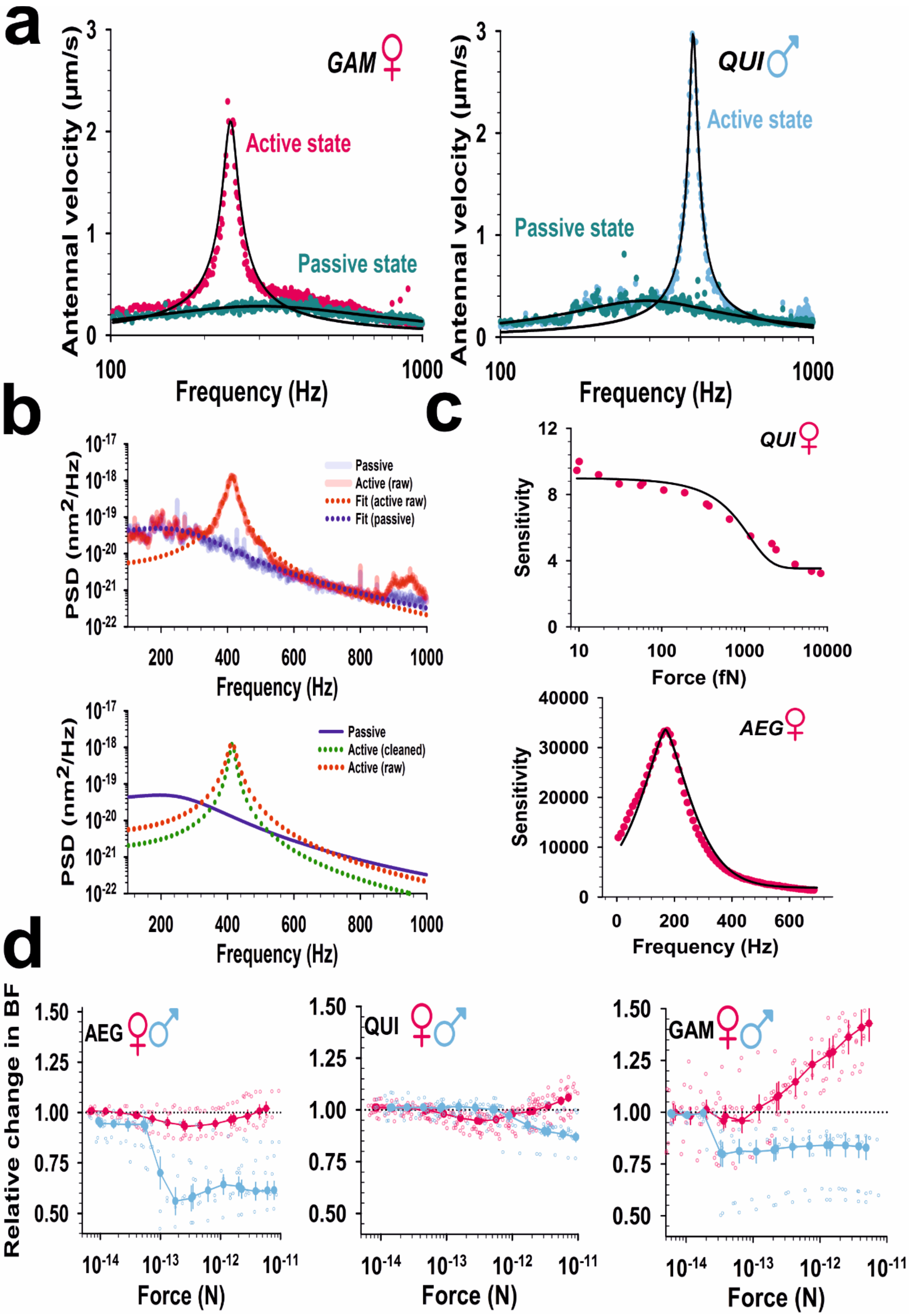

Supplementary Figure 1. Auxiliary data illustrating experimental procedures

a Free, unstimulated fluctuations in both active (red dots/ blue dots) and passive (green dots) states over a frequency range of 100 to 1000Hz for an *An. gambiae* (GAM) female (left) and a *Cx. quinquefasciatus* (QUI) male (right). Individual data points represent Fourier-transformed velocity amplitudes whilst solid black lines show the velocity amplitude function fits for each state.

b (top) Free, unstimulated fluctuations in both active (faded red line) and passive (faded blue line) states for a single, quiescent antenna of a *Cx. quinquefasciatus* male over a frequency range of 100 to 1000Hz with associated fits (active, red dotted line; passive, blue dotted line) as used for the extraction of here reported system parameters; please note that there remain minor discrepancies between fit and data. (bottom) Fits for active antenna before (raw data, red dotted line) and after (cleaned data, green dotted line) subtraction of the passive system, as well as fit for passive system (blue solid line) for the same male. Maximum possible errors due to fit discrepancies are ~25%. Such small deviations do not affect any of the paper's conclusions.

c (top) Changes in mechanical sensitivity in response to increases in white noise stimulus intensity for an individual *Cx. quinquefasciatus* (QUI) female. Each point represents the mechanical sensitivity at an individual attenuation of the stimulus and the black line represents the best fit of a sigmoid function. (bottom) Changes in mechanical sensitivity in response to increases in pure tone stimulus frequency with a Gaussian fit (solid black line) to individual sensitivity data points for an *Ae. aegypti* (AEG) female. Each point represents the mechanical sensitivity at an individual attenuation of the stimulus.

d Changes in median flagellar best frequency (BF) in response to increasing intensity of white noise stimulation female (red) and male (blue) *Ae. aegypti* (AEG), *Cx. quinquefasciatus* (QUI) and *An. gambiae* (GAM). Each point represents the relative change in flagellar best frequency at an individual attenuation of the stimulus. Dashed horizontal line represents line of unity whilst the solid lines represent relative changes to female (red solid line) and male (blue solid line) flagellar best frequencies. Error bars represent ± SEM. Sample sizes: *Aedes aegypti* females = 7; *Aedes aegypti* males = 7; *Culex quinquefasciatus* females = 13; *Culex quinquefasciatus* males = 13; *Anopheles gambiae* females = 9; *Anopheles gambiae* males = 7.

Supplementary Figure 2

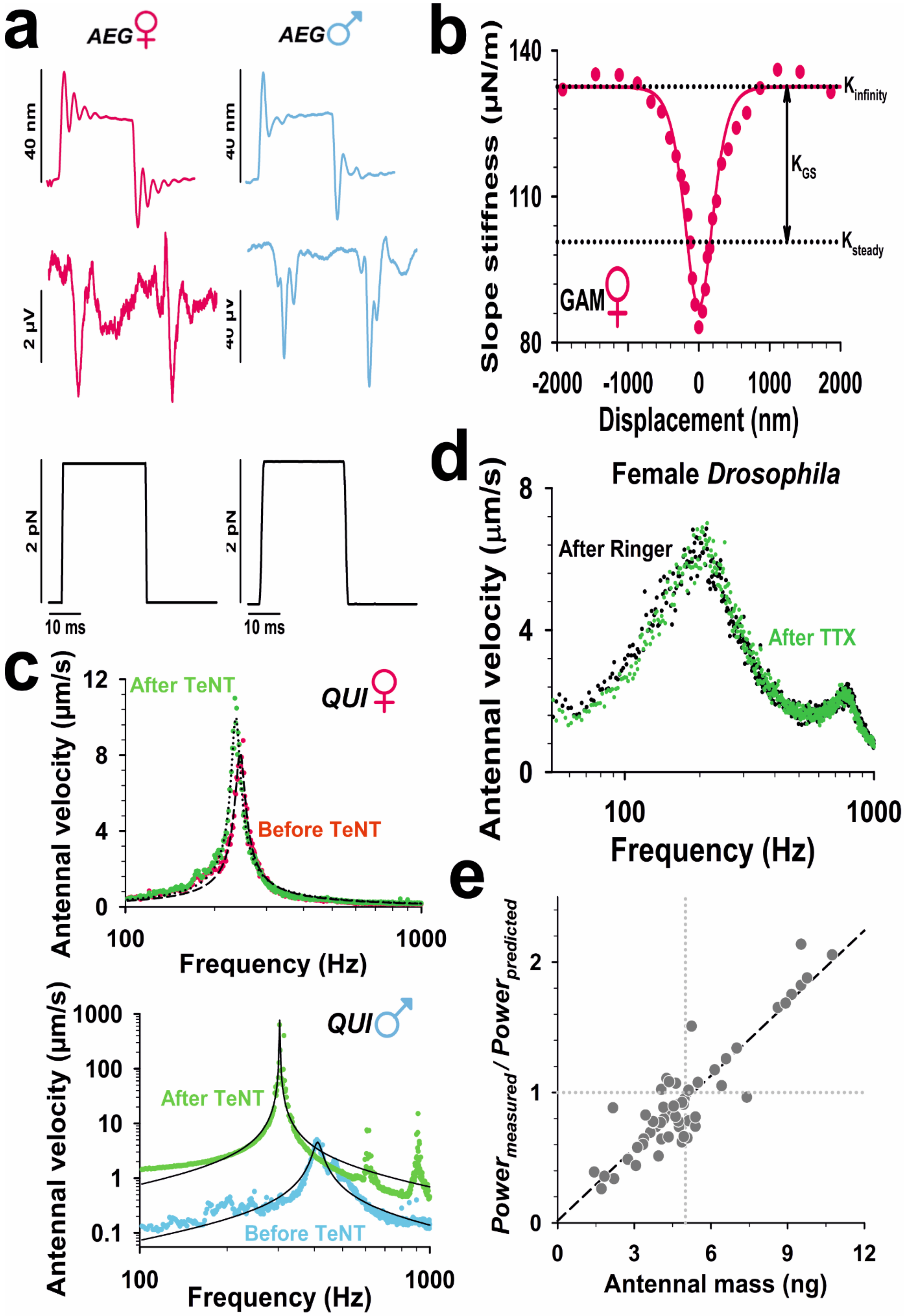

Supplementary Figure 2. Auxiliary data illustrating experimental procedures

a Flagellar displacement (top) and antennal nerve response amplitude (middle) in response to the corresponding force step (bottom) for female (left) and male (right) *Ae. aegypti* (AEG).

b Diagrammatic representation of $K_{INFINITY}$, K_{STEADY} and K_{GS}. Slope stiffness values (red dots) are for *An. gambiae* females. Solid red line shows the best fit of the single transducer population model to the stiffness data.

c Free, unstimulated fluctuations both before (i.e. following ringer injection, red or blue dots) and after (green dots) TeNT injection over a frequency range of 100 to 1000Hz for a female (top) and male (bottom) *Cx. quinquefasciatus* (QUI). Individual data points represent Fourier-transformed velocity amplitudes whilst solid black lines show the velocity amplitude function fits for each state.

d Free, unstimulated fluctuations both before (i.e. following ringer injection, black dots) and after (green dots) TTX injection over a frequency range of 100 to 1000Hz for a female *Drosophila melanogaster*. Individual data points represent Fourier-transformed velocity amplitudes whilst solid black lines show the velocity amplitude function fits for each recording.

e Ratio of measured and predicted energy gains (as calculated using the previously reported calculation method and the procedure described here in the materials and methods) for *Drosophila melanogaster*. Individual data points represent individual *Drosophila*. Note the strong correlation between apparent antennal mass and the power gain ratio; ratios of 1 occur at ~5ng (the estimated apparent antennal mass used previously for *Drosophila*) as represented by grey dotted lines.

Supplementary Figure 3

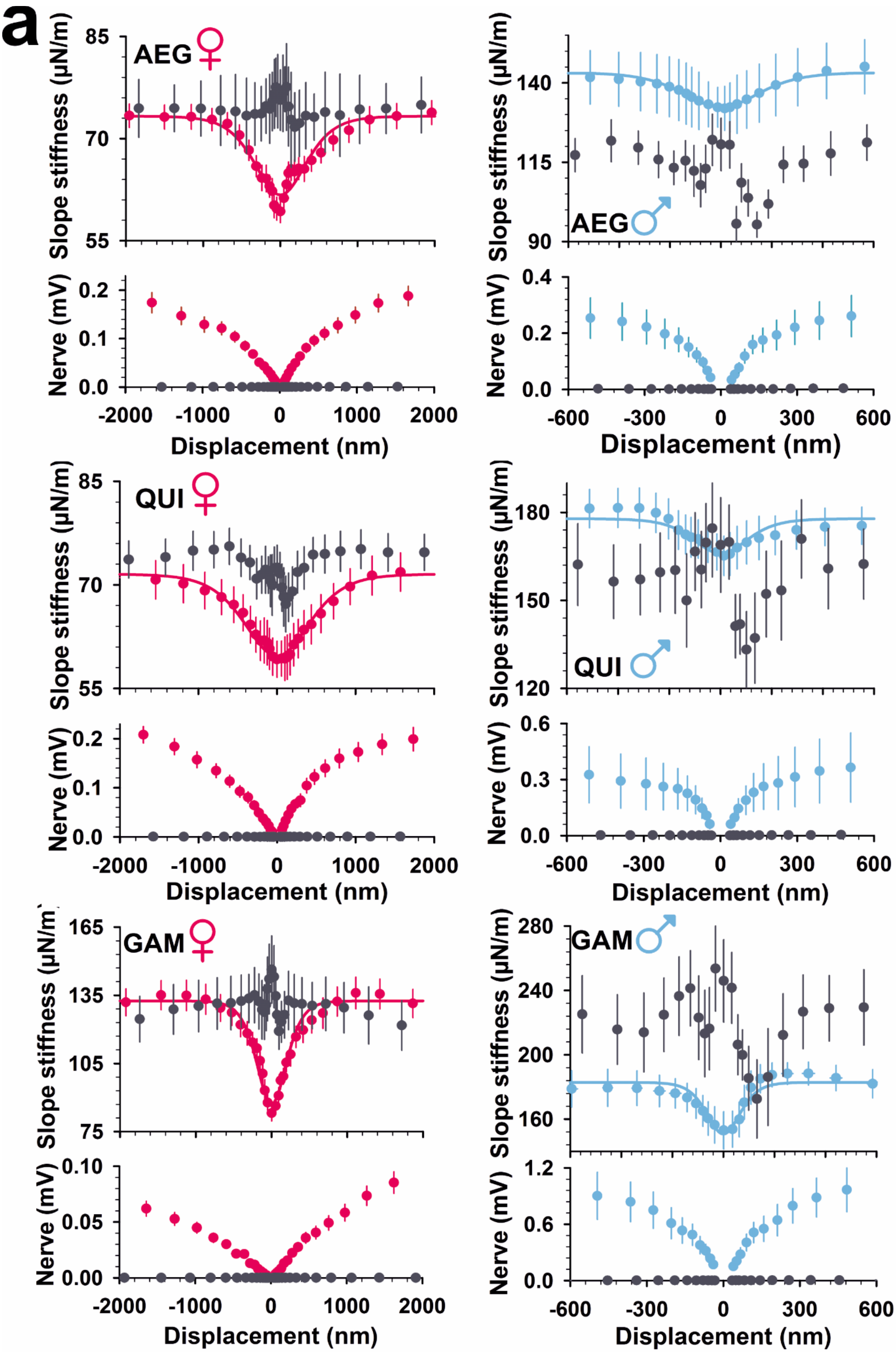

Supplementary Figure 3. Changes in slope stiffness and CAP magnitude following pymetrozine exposure

a Median receiver slope stiffness and nerve responses in response to flagellar displacements for female and male *Ae. aegypti* (AEG), *Cx. quinquefasciatus* (QUI) and *An. gambiae* (GAM) both before (females red, males blue) and after (grey) pymetrozine exposure. Solid lines show the best fit of the single transducer population model to the stiffness data. Error bars represent ± SEM. Sample sizes (before and after pymetrozine): *Ae. aegypti* females = 21/ 7; *Ae. aegypti* males = 18/ 7; *Cx. quinquefasciatus* females = 17/ 7; *Cx. quinquefasciatus* males = 15/ 7; *An. gambiae* females = 18/ 7; *An. gambiae* males = 11/ 7.

Supplementary Figure 4

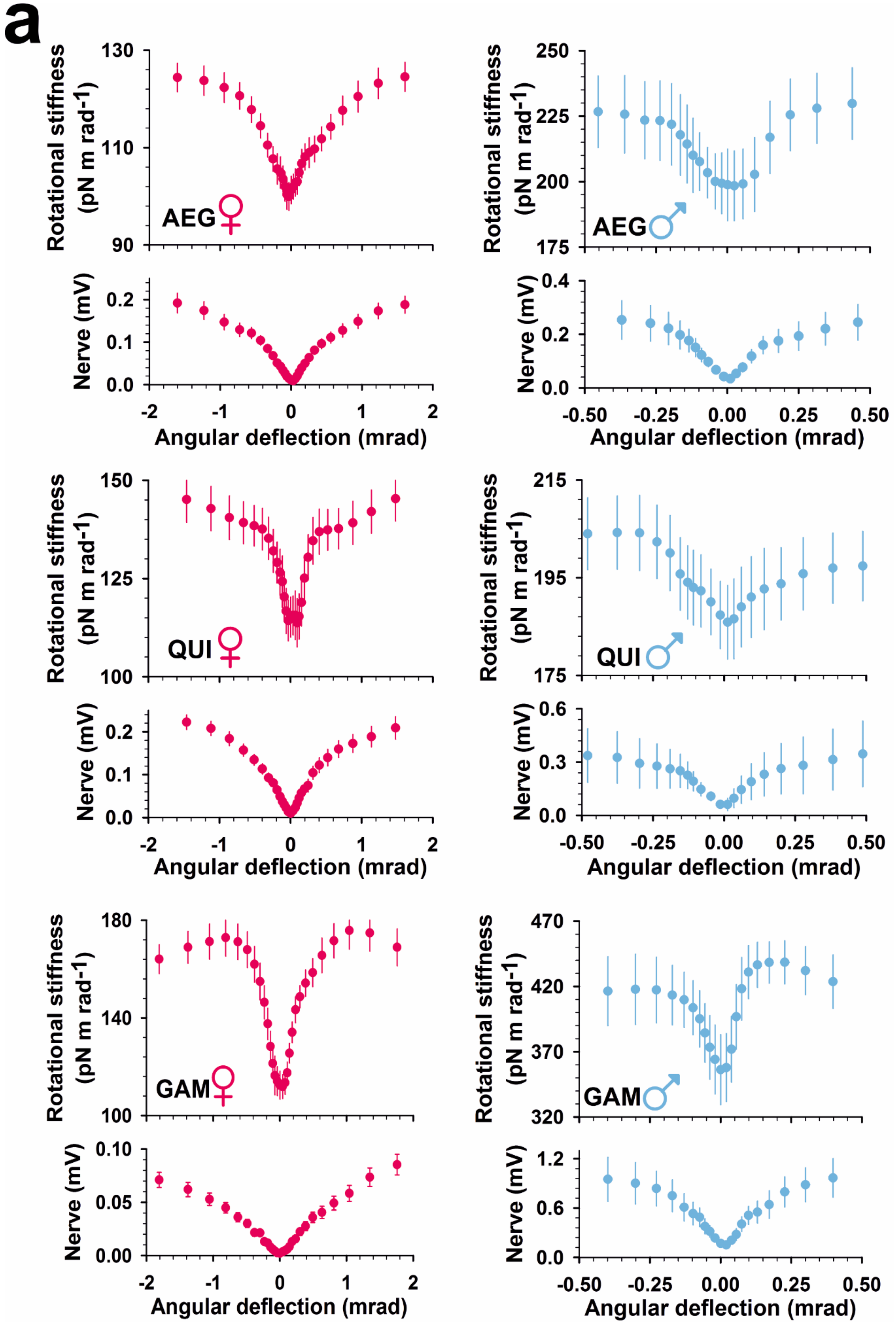

Supplementary Figure 4. Changes in slope stiffness and CAP magnitude in response to angular deflections of the flagellum

a Median receiver slope stiffness and nerve responses in response to flagellar displacements in terms of angular deflections for female (red) and male (blue) *Ae. aegypti* (AEG), *Cx. quinquefasciatus* (QUI) and *An. gambiae* (GAM). Error bars represent ± SEM. Sample sizes: *Ae. aegypti* females = 21; *Ae. aegypti* males = 18; *Cx. quinquefasciatus* females = 17; *Cx. quinquefasciatus* males = 15; *An. gambiae females* = 18; *An. gambiae* males = 11.

Supplementary Figure 5

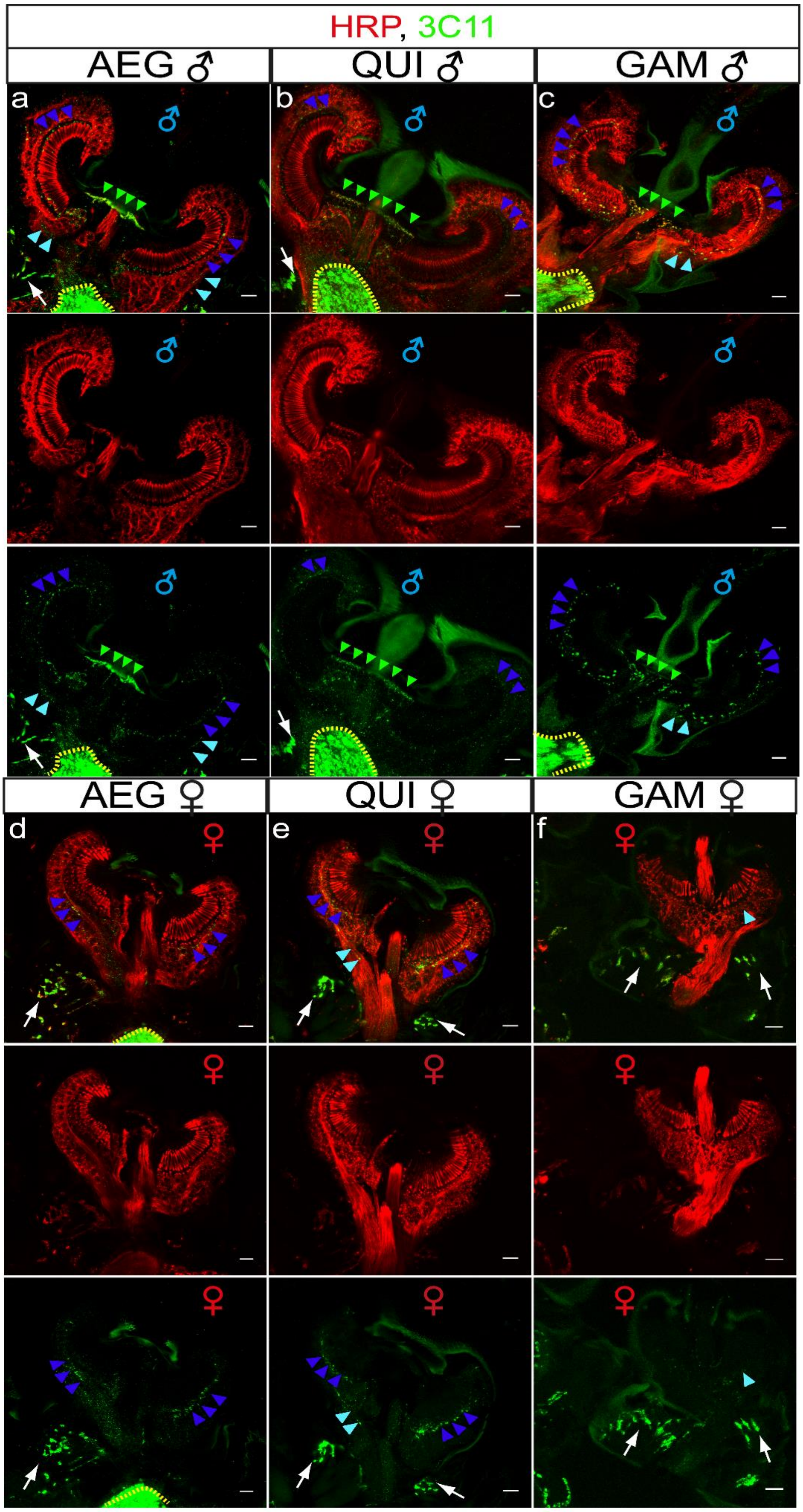

Supplementary Figure 5: Sexual dimorphism in the auditory efferent innervation of all three mosquito species

JO horizontal sections were stained with the presynaptic marker 3C11 (anti-synapsin, green) to label auditory efferent fibres and counterstained with the neuronal marker anti-HRP (red). *Ae. aegypti* (AEG), *Cx. quinquefasciatus* (QUI) and *An. gambiae* (GAM).

a - c Male mosquito JO of all three species present an extensive efferent innervation pattern in the basal plate (green arrowheads), base of auditory cilia (dark blue arrowheads), intermingled among somata (light blue arrowheads) and in the auditory nerve (yellow dash line). Neuronal marker anti-HRP staining (red, middle panel); presynaptic marker 3C11 (green, bottom panel).

d-f Female mosquito JO staining. **d-e** In AEG and QUI females the efferent fibres innervate the base of the auditory cilia (dark blue arrowheads) and somata region (light blue arrowheads). **f** Efferent innervation in GAM females is limited to dispersed punctae intermingled among the somata (light blue arrowhead). Neuronal marker anti-HRP staining (red, middle panel); presynaptic marker 3C11 (green, bottom panel).

3C11 also stains motoneuronal innervation of muscles in the scape (arrow). Scale bar: 10µm.

Supplementary Table 1. Measurements of flagellar length

Median values for total flagellar length as well as the distance between the laser focus point and the pedicel for female and male *Ae. aegypti* (AEG), *Cx. quinquefasciatus* (QUI) and *An. gambiae* (GAM) (SEM is given in brackets).

	AEG ♀	*AEG* ♂	*QUI* ♀	*QUI* ♂	*GAM* ♀	*GAM* ♂
Sample size	8	10	23	16	9	22
Length from laser focus point to base (µm)	1282.30 (51.0)	1244.11 (22.0)	1447.17 (36.1)	1066.57 (39.7)	1119.26 (33.3)	1551.68 (42.5)
Total length (µm)	1795.83 (64.6)	1630.86 (46.0)	1863.21 (25.2)	1400.99 (26.0)	1548.03 (42.7)	1736.68 (68.5)

Supplementary Table 2. Comparisons between spontaneous and induced SOs

Median values obtained from harmonic oscillator fits for *An. gambiae* males in either the spontaneous or pharmacologically induced SO states (SEM is given in brackets); these include the best frequency and tuning sharpness (Q) of the flagellum. The pharmacologically induced SO state includes data from both TTX and TeNT injection experiments.

	Spontaneous SO state	Induced SO state
Sample size	10	15
Best frequency/Hz	336.51<br>(7.9)	332.04<br>(5.8)
Tuning sharpness Q	496.30<br>(506.1)	355.79<br>(895.09)

Supplementary Table 3. ANOVA values for statistical comparisons of power gain

H = 32.703 with 5 degrees of freedom (P<0.001). For comparisons found to be statistically significant (P<0.05), the group with the significantly greater value is highlighted in bold.

Comparison	Difference on Ranks	Q	P
***Cx. quinquefasciatus* females** vs *Cx. quinquefasciatus* males	56.911	4.284	**<0.001**
***Cx. quinquefasciatus* females** vs *Ae. aegypti* females	49.721	3.849	**0.002**
***Cx. quinquefasciatus* females** vs *Ae. aegypti* males	47.321	3.475	**0.008**
***Cx. quinquefasciatus* females** vs *An. gambiae* females	70.598	5.393	**<0.001**
***Cx. quinquefasciatus* females** vs *An. gambiae* males	55.083	3.745	**0.003**
Cx. quinquefasciatus males vs *Ae. aegypti* females	7.190	0.572	>0.05
Cx. quinquefasciatus males vs *Ae. aegypti* males	9.590	0.722	>0.05
Cx. quinquefasciatus males vs *An. gambiae* females	13.687	1.074	>0.05
Cx. quinquefasciatus males vs *An. gambiae* males	1.828	0.127	>0.05
Ae. aegypti females vs *Ae. aegypti* males	2.400	0.186	>0.05
Ae. aegypti females vs *An. gambiae* females	20.877	1.689	>0.05
Ae. aegypti females vs *An. gambiae* males	5.362	0.381	>0.05
Ae. aegypti males vs *An. gambiae* females	23.277	1.778	>0.05
Ae. aegypti males vs *An. gambiae* males	7.762	0.528	>0.05
An. gambiae females vs *An. gambiae* males	15.515	1.091	>0.05

Supplementary Table 4. ANOVA values for statistical comparisons of female *Ae. aegypti* flagellar best frequency, tuning sharpness and power gain between different states (active, passive, Pymetrozine, Ringer, TTX and TeNT)

For comparisons found to be statistically significant (P<0.05), the group with the significantly greater value is highlighted in bold.

	Difference on Ranks	Q	P	H value
Best frequency comparisons				
Active vs Passive	-	-	>0.05	3.288 with 2 degrees of freedom (P >0.05)
Active vs Pymetrozine	-	-	>0.05	
Passive vs Pymetrozine	-	-	>0.05	
Ringer vs TTX	-	-	>0.05	3.950 with 2 degrees of freedom (P=0.139)
Ringer vs TeNT	-	-	>0.05	
TTX vs TeNT	-	-	>0.05	
Tuning sharpness comparisons				
**Active** vs Passive	68.963	8.848	**<0.001**	91.053 with 2 degrees of freedom (P<0.001)
**Active** vs Pymetrozine	52.996	6.856	**<0.001**	
Passive vs Pymetrozine	15.967	1.887	>0.05	
Ringer vs TTX	-	-	>0.05	2.851 with 2 degrees of freedom (P>0.05)
Ringer vs TeNT	-	-	>0.05	
TTX vs TeNT	-	-	>0.05	
Power gain comparisons				
Ringer vs TTX	2.210	0.319	>0.05	37.951 with 3 degrees of freedom (P<0.001)
Ringer vs TeNT	2.165	0.312	>0.05	
TTX vs TeNT	0.0455	0.0057	>0.05	
**Ringer** vs Pymetrozine	31.595	5.499	**<0.001**	

Supplementary Table 5. ANOVA values for statistical comparisons of male *Ae. aegypti* flagellar best frequency, tuning sharpness and power gain between different states (active, passive, Pymetrozine, Ringer, TTX and TeNT)

For comparisons found to be statistically significant (P<0.05), the group with the significantly greater value is highlighted in bold.

	Difference on Ranks	Q	P	H value
Best frequency comparisons				
**Active** vs Passive	44.403	6.778	**<0.001**	66.294 with 2 degrees of freedom (P<0.001)
**Active** vs Pymetrozine	43.281	6.755	**<0.001**	
Passive vs Pymetrozine	1.122	0.156	>0.05	
**Ringer** vs TTX	13.907	2.826	**0.014**	18.438 with 2 degrees of freedom (P <0.001)
**Ringer** vs TeNT	17.256	3.849	**<0.001**	
TTX vs TeNT	3.349	0.583	>0.05	
Tuning sharpness comparisons				
**Active** vs Passive	50.669	7.929	**<0.001**	H = 66.359 with 2 degrees of freedom (P<0.001)
**Active** vs Pymetrozine	32.158	4.850	**<0.001**	
Passive vs **Pymetrozine**	18.511	2.590	**0.029**	
Ringer vs **TTX**	15.467	3.219	**0.009**	21.362 with 2 degrees of freedom (P<0.001)
Ringer vs **TeNT**	22.689	4.264	<**0.001**	
TTX vs TeNT	8.222	1.246	0.638	
Power gain comparisons				
Ringer vs **TTX**	15.762	3.297	**0.009**	35.621 with 3 degrees of freedom (P<0.001)
Ringer vs **TeNT**	16.335	3.921	**0.007**	
TTX vs TeNT	1.625	0.237	>0.05	
**Ringer** vs Pymetrozine	30.200	5.031	**<0.001**	

Supplementary Table 6. ANOVA values for statistical comparisons of female *Cx. quinquefasciatus* flagellar best frequency, tuning sharpness and power gain between different states (active, passive, Pymetrozine, Ringer, TTX and TeNT)

For comparisons found to be statistically significant (P<0.05), the group with the significantly greater value is highlighted in bold.

	Difference on Ranks	Q	P	H value
Best frequency comparisons				
Active vs Passive	-	-	>0.05	3.320 with 2 degrees of freedom (P>0.05)
Active vs Pymetrozine	-	-	>0.05	
Passive vs Pymetrozine	-	-	>0.05	
Ringer vs TTX	-	-	>0.05	2.950 with 2 degrees of freedom (P= 0.229)
Ringer vs TeNT	-	-	>0.05	
TTX vs TeNT	-	-	>0.05	
Tuning sharpness comparisons				
**Active** vs Passive	35.792	5.200	**<0.001**	52.217 with 2 degrees of freedom (P<0.001)
**Active** vs Pymetrozine	37.571	5.407	**<0.001**	
Passive vs **Pymetrozine**	27.364	3.550	**0.001**	
Ringer vs **TTX**	13.222	2.859	**0.013**	10.626 with 2 degrees of freedom (P= 0.005)
Ringer vs **TeNT**	13.684	2.932	**0.011**	
TTX vs TeNT	0.571	0.140	>0.05	
Power gain comparisons				
Ringer vs **TTX**	20.237	2.896	**0.030**	46.239 with 3 degrees of freedom (P<0.001)
Ringer vs **TeNT**	19.425	2.735	**0.042**	
TTX vs TeNT	0.614	0.0729	>0.05	
**Ringer** vs Pymetrozine	34.603	5.254	**<0.001**	

Supplementary Table 7. ANOVA values for statistical comparisons of male *Cx. quinquefasciatus* flagellar best frequency, tuning sharpness and power gain between different states (active, passive, Pymetrozine, Ringer, TTX and TeNT)

For comparisons found to be statistically significant (P<0.05), the group with the significantly greater value is highlighted in bold.

	Difference on Ranks	Q	P	H value
Best frequency comparisons				
**Active** vs Passive	52.403	7.062	**<0.001**	74.451 with 2 degrees of freedom (P<0.001)
**Active** vs Pymetrozine	55.429	7.592	**<0.001**	
Passive vs Pymetrozine	3.026	0.391	>0.05	
**Ringer** vs TTX	19.898	4.185	**<0.001**	31.739 with 2 degrees of freedom (P<0.001)
**Ringer** vs TeNT	25.550	5.274	**<0.001**	
TTX vs TeNT	5.652	1.133	0.771	
Tuning sharpness comparisons				
**Active** vs Passive	57.063	7.690	**<0.001**	71.394 with 2 degrees of freedom (P<0.001)
**Active** vs Pymetrozine	47.967	6.570	**<0.001**	
Passive vs **Pymetrozine**	9.096	1.175	0.720	
Ringer vs **TTX**	20.247	4.258	**<0.001**	30.784 with 2 degrees of freedom (P <0.001)
Ringer vs **TeNT**	24.790	5.117	**<0.001**	
TTX vs TeNT	4.543	0.911	>0.05	
Power gain comparisons				
Ringer vs **TTX**	27.103	3.731	**<0.001**	68.782 with 3 degrees of freedom (P<0.001)
Ringer vs **TeNT**	31.246	4.184	**<0.001**	
TTX vs TeNT	4.143	0.486	>0.05	
**Ringer** vs Pymetrozine	24.754	3.764	**<0.001**	

Supplementary Table 8. ANOVA values for statistical comparisons of female *An. gambiae* flagellar best frequency, tuning sharpness and power gain between different states (active, passive, Pymetrozine, Ringer, TTX and TeNT)

For comparisons found to be statistically significant (P<0.05), the group with the significantly greater value is highlighted in bold.

	Difference on Ranks	Q	P	H value
Best frequency comparisons				
Active vs **Passive**	49.892	7.320	**<0.001**	65.799 with 2 degrees of freedom (P<0.001)
Active vs **Pymetrozine**	45.260	6.190	**<0.001**	
Passive vs Pymetrozine	4.632	0.603	>0.05	
Ringer vs TTX	-	-	>0.05	0.950 with 2 degrees of freedom (P= 0.622)
Ringer vs TeNT	-	-	>0.05	
TTX vs TeNT	-	-	>0.05	
Tuning sharpness comparisons				
**Active** vs Passive	43.545	6.389	**<0.001**	40.826 with 2 degrees of freedom (P<0.001)
**Active** vs Pymetrozine	25.007	3.255	**0.003**	
Passive vs **Pymetrozine**	18.538	2.535	**0.034**	
Ringer vs TTX	-	-	>0.05	0.0554 with 2 degrees of freedom (P= 0.973)
Ringer vs TeNT	-	-	>0.05	
TTX vs TeNT	-	-	>0.05	
Power gain comparisons				
Ringer vs TTX	1.811	0.274	>0.05	H = 28.924 with 3 degrees of freedom (P<0.001)
Ringer vs TeNT	1.625	0.239	>0.05	
TTX vs TeNT	3.436	0.447	>0.05	
**Ringer** vs Pymetrozine	29.042	4.772	**<0.001**	

Supplementary Table 9. ANOVA values for statistical comparisons of male *An. gambiae* flagellar best frequency, tuning sharpness and power gain between different states (active, passive, Pymetrozine, Ringer, TTX and TeNT)

For comparisons found to be statistically significant (P<0.05), the group with the significantly greater value is highlighted in bold.

	Difference on Ranks	Q	P	H value
Best frequency comparisons				
**Active** vs Passive	40.018	7.006	**<0.001**	52.822 with 2 degrees of freedom (P<0.001)
**Active** vs Pymetrozine	27.700	4.565	**<0.001**	
Passive vs Pymetrozine	12.318	1.904	0.171	
**Ringer** vs TTX	13.940	3.166	**0.005**	20.839 with 2 degrees of freedom (P<0.001)
**Ringer** vs TeNT	16.993	4.049	**<0.001**	
TTX vs TeNT	3.054	0.592	>0.05	
Tuning sharpness comparisons				
**Active** vs Passive	41.536	7.271	**<0.001**	55.515 with 2 degrees of freedom (P<0.001)
**Active** vs Pymetrozine	26.622	4.388	**<0.001**	
Passive vs Pymetrozine	14.914	2.306	0.063	
**Ringer** vs TTX	15.286	3.472	**0.002**	24.818 with 2 degrees of freedom (P<0.001)
**Ringer** vs TeNT	18.500	4.408	**<0.001**	
TTX vs TeNT	3.214	0.624	>0.05	
Power gain comparisons				
Ringer vs **TTX**	15.950	3.033	**0.008**	36.452 with 3 degrees of freedom (P<0.001)
Ringer vs **TeNT**	27.643	4.738	**<0.001**	
TTX vs TeNT	1.607	0.253	>0.05	
**Ringer** vs Pymetrozine	29.250	5.224	**<0.001**	

Supplementary Table 10. ANOVA values for statistical comparisons of female mosquito CAP magnitude

H = 23.779 with 2 degrees of freedom (P<0.001). For comparisons found to be statistically significant (P<0.05), the group with the significantly greater value is highlighted in bold.

Comparison	Difference on Ranks	Q	P
Cx. quinquefasciatus vs *An. gambiae*	21.778	3.936	**<0.001**
Cx. quinquefasciatus vs *Ae. aegypti*	2.230	0.418	1.000
Ae. aegypti vs *An. gambiae*	24.008	4.503	**<0.001**

Supplementary Table 11. ANOVA values for statistical comparisons of estimated number of transducer channels, *N*

H = 51.650 with 5 degrees of freedom (P<0.001). For comparisons found to be statistically significant (P<0.05), the group with the significantly greater value is highlighted in bold.

Comparison	Difference on Ranks	Q	P
***Cx. quinquefasciatus* females** vs *Cx. quinquefasciatus* males	55.689	5.384	**<0.001**
Cx. quinquefasciatus females vs *Ae. aegypti* females	6.222	0.655	>0.05
Cx. quinquefasciatus females vs *Ae. aegypti* males	23.056	2.338	0.291
Cx. quinquefasciatus females vs *An. gambiae* females	5.817	0.581	>0.05
***Cx. quinquefasciatus* females** vs *An. gambiae* males	37.812	3.511	**0.007**
Cx. quinquefasciatus males vs ***Ae. aegypti* females**	49.467	4.945	**<0.001**
Cx. quinquefasciatus males vs *Ae. aegypti* males	22.633	2.235	>0.05
Cx. quinquefasciatus males vs ***An. gambiae* females**	61.506	5.868	**<0.001**
Cx. quinquefasciatus males vs *An. gambiae* males	17.877	1.594	>0.05
***Ae. aegypti* females** vs *Ae. aegypti* males	36.833	3.471	**0.008**
Ae. aegypti females vs *An. gambiae* females	12.039	1.247	>0.05
***Ae. aegypti* females** vs *An. gambiae* males	31.590	3.025	**0.037**
Ae. aegypti males vs *An. gambiae* females	28.873	2.885	>0.05
Ae. aegypti males vs *An. gambiae* males	14.756	1.370	>0.05
***An. gambiae* females** vs *An. gambiae* males	43.629	4.002	**<0.001**

Supplementary Table 12. ANOVA values for statistical comparisons of single channel gating force, *z*

H = 53.987 with 5 degrees of freedom (P<0.001). For comparisons found to be statistically significant (P<0.05), the group with the significantly greater value is highlighted in bold.

Comparison	Difference on Ranks	Q	P
Cx. quinquefasciatus females vs ***Cx. quinquefasciatus* males**	63.011	6.091	**<0.001**
Cx. quinquefasciatus females vs *Ae. aegypti* females	9.563	1.006	>0.05
Cx. quinquefasciatus females vs *Ae. aegypti* males	26.278	2.664	0.116
Cx. quinquefasciatus females vs ***An. gambiae* females**	31.866	3.184	**0.022**
Cx. quinquefasciatus females vs ***An. gambiae* males**	51.739	4.804	**<0.001**
***Cx. quinquefasciatus* males** vs *Ae. aegypti* females	53.448	5.343	**<0.001**
Cx. quinquefasciatus males vs *Ae. aegypti* males	16.733	1.771	>0.05
***Cx. quinquefasciatus* males** vs *An. gambiae* females	31.145	2.971	**0.044**
Cx. quinquefasciatus males vs *An. gambiae* males	11.272	1.005	>0.05
Ae. aegypti females vs *Ae. aegypti* males	16.714	1.759	>0.05
Ae. aegypti females vs ***An. gambiae* females**	32.303	3.210	**0.020**
***Ae. aegypti* females** vs *An. gambiae* males	42.176	4.039	**<0.001**
Ae. aegypti males vs *An. gambiae* females	5.588	0.558	>0.05
Ae. aegypti males vs *An. gambiae* males	25.462	2.364	0.271
An. gambiae females vs ***An. gambiae* males**	49.873	4.653	**<0.001**

Supplementary Table 13. ANOVA values for statistical comparisons of K_{STEADY}

H = 80.409 with 5 degrees of freedom (P<0.001). For comparisons found to be statistically significant (P<0.05), the group with the significantly greater value is highlighted in bold.

Comparison	Difference on Ranks	Q	P
Cx. quinquefasciatus females vs ***Cx. quinquefasciatus*** **males**	65.333	6.316	**<0.001**
Cx. quinquefasciatus females vs *Ae. aegypti* females	4.952	0.521	>0.05
Cx. quinquefasciatus females vs ***Ae. aegypti*** **males**	48.778	4.946	**<0.001**
Cx. quinquefasciatus females vs ***An. gambiae*** **females**	39.725	3.970	**0.001**
Cx. quinquefasciatus females vs ***An. gambiae*** **males**	65.205	6.055	**<0.001**
Cx. quinquefasciatus **males** vs *Ae. aegypti* females	60.381	6.036	**<0.001**
Cx. quinquefasciatus males vs *Ae. aegypti* males	16.556	1.600	>0.05
Cx. quinquefasciatus males vs *An. gambiae* females	25.608	2.443	0.218
Cx. quinquefasciatus males vs ***An. gambiae*** **males**	36.128	3.810	**0.002**
Ae. aegypti females vs ***Ae. aegypti*** **males**	43.825	4.611	**<0.001**
Ae. aegypti females vs ***An. gambiae*** **females**	34.773	3.602	**0.005**
Ae. aegypti females vs ***An. gambiae*** **males**	60.253	5.770	**<0.001**
Ae. aegypti males vs *An. gambiae* females	9.052	0.905	>0.05
Ae. aegypti males vs ***An. gambiae*** **males**	36.427	3.825	**0.002**
An. gambiae females vs ***An. gambiae*** **males**	35.480	3.737	**0.004**

Supplementary Table 14. ANOVA values for statistical comparisons of Kinfinity

H = 83.421 with 5 degrees of freedom (P<0.001). For comparisons found to be statistically significant (P<0.05), the group with the significantly greater value is highlighted in bold.

Comparison	Difference on Ranks	Q	P
Cx. quinquefasciatus females vs ***Cx. quinquefasciatus* males**	73.289	7.085	**<0.001**
Cx. quinquefasciatus females vs *Ae. aegypti* females	10.413	1.096	>0.05
Cx. quinquefasciatus females vs ***Ae. aegypti* males**	51.556	5.227	**<0.001**
Cx. quinquefasciatus females vs ***An. gambiae* females**	41.026	4.100	**<0.001**
Cx. quinquefasciatus females vs ***An. gambiae* males**	64.325	5.973	**<0.001**
***Cx. quinquefasciatus* males** vs *Ae. aegypti* females	62.876	6.286	**<0.001**
Cx. quinquefasciatus males vs *Ae. aegypti* males	21.733	2.101	0.535
***Cx. quinquefasciatus* males** vs *An. gambiae* females	32.263	3.078	**0.031**
Cx. quinquefasciatus males vs ***An. gambiae* males**	31.964	3.026	**0.028**
Ae. aegypti females vs ***Ae. aegypti* males**	41.143	4.329	**<0.001**
Ae. aegypti females vs ***An. gambiae* females**	30.613	3.171	**0.023**
Ae. aegypti females vs ***An. gambiae* males**	53.912	5.163	**<0.001**
Ae. aegypti males vs *An. gambiae* females	10.529	1.052	>0.05
Ae. aegypti males vs ***An. gambiae* males**	32.769	3.154	**0.026**
An. gambiae females vs ***An. gambiae* males**	43.299	4.437	**<0.001**

Supplementary Table 15. ANOVA values for statistical comparisons of K_{GS}

H = 78.201 with 5 degrees of freedom (P<0.001). For comparisons found to be statistically significant (P<0.05), the group with the significantly greater value is highlighted in bold.

Comparison	Difference on Ranks	Q	P
Cx. quinquefasciatus females vs ***Cx. quinquefasciatus* males**	39.867	3.854	**0.002**
Cx. quinquefasciatus females vs *Ae. aegypti* females	5.381	0.566	>0.05
Cx. quinquefasciatus females vs ***Ae. aegypti* males**	46.444	4.709	**<0.001**
Cx. quinquefasciatus females vs ***An. gambiae* females**	42.529	4.250	**<0.001**
Cx. quinquefasciatus females vs ***An. gambiae* males**	66.385	6.164	**<0.001**
***Cx. quinquefasciatus* males** vs *Ae. aegypti* females	45.248	4.523	**<0.01**
Cx. quinquefasciatus males vs *Ae. aegypti* males	6.578	0.636	>0.05
Cx. quinquefasciatus males vs *An. gambiae* females	2.663	0.254	>0.05
Cx. quinquefasciatus males vs ***An. gambiae* males**	46.518	4.695	**<0.001**
Ae. aegypti females vs ***Ae. aegypti* males**	51.825	5.453	**<0.001**
Ae. aegypti females vs ***An. gambiae* females**	47.910	4.963	**<0.001**
Ae. aegypti females vs ***An. gambiae* males**	71.766	6.873	**<0.001**
Ae. aegypti males vs *An. gambiae* females	3.915	0.391	>0.05
Ae. aegypti males vs ***An. gambiae* males**	40.940	3.992	**<0.001**
An. gambiae females vs ***An. gambiae* males**	43.855	4.328	**<0.001**

Supplementary Table 16. ANOVA values for statistical comparisons of the extent of non-linearity

H = 60.628 with 5 degrees of freedom (P<0.001). For comparisons found to be statistically significant (P<0.05), the group with the significantly greater value is highlighted in bold.

Comparison	Difference on Ranks	Q	P
***Cx. quinquefasciatus* females vs** *Cx. quinquefasciatus* males	30.500	2.948	**0.048**
Cx. quinquefasciatus females vs *Ae. aegypti* females	2.262	0.238	>0.05
***Cx. quinquefasciatus* females** vs *Ae. aegypti* males	31.944	3.239	**0.018**
Cx. quinquefasciatus females vs ***An. gambiae* females**	33.971	3.395	**0.01**
Cx. quinquefasciatus females vs *An. gambiae* males	24.115	2.239	0.377
Cx. quinquefasciatus males vs *Ae. aegypti* females	28.238	2.823	0.071
Cx. quinquefasciatus males vs *Ae. aegypti* males	1.444	0.140	>0.05
Cx. quinquefasciatus males vs ***An. gambiae* females**	64.471	6.151	**<0.001**
Cx. quinquefasciatus males vs *An. gambiae* males	36.385	3.869	**0.002**
***Ae. aegypti* females** vs *Ae. aegypti* males	29.683	3.123	**0.027**
Ae. aegypti females vs ***An. gambiae* females**	36.232	3.753	**0.003**
Ae. aegypti females vs *An. gambiae* males	21.853	2.093	0.545
Ae. aegypti males vs ***An. gambiae* females**	65.915	6.587	**<0.001**
Ae. aegypti males vs *An. gambiae* males	37.829	3.987	**<0.001**
***An. gambiae* females** vs *An. gambiae* males	58.086	5.328	**<0.001**

Supplementary Table 17. ANOVA values for statistical comparisons of the estimated *CAP_{50}* values

H = 82.657 with 5 degrees of freedom (P<0.001). For comparisons found to be statistically significant (P<0.05), the group with the significantly greater value is highlighted in bold.

Comparison	Difference on Ranks	Q	P
***Cx. quinquefasciatus* females** vs *Cx. quinquefasciatus* males	30.521	2.987	**0.042**
Cx. quinquefasciatus females vs *Ae. aegypti* females	16.381	1.704	>0.05
***Cx. quinquefasciatus* females** vs *Ae. aegypti* males	30.686	3.046	**0.035**
Cx. quinquefasciatus females vs ***An. gambiae* females**	31.725	3.150	**0.025**
***Cx. quinquefasciatus* females** vs *An. gambiae* males	37.083	3.367	**0.011**
Cx. quinquefasciatus males vs ***Ae. aegypti* females**	46.902	4.971	**<0.001**
Cx. quinquefasciatus males vs *Ae. aegypti* males	0.165	0.0167	>0.05
Cx. quinquefasciatus males vs ***An. gambiae* females**	62.246	6.285	**<0.001**
Cx. quinquefasciatus males vs *An. gambiae* males	6.563	0.604	>0.05
***Ae. aegypti* females** vs *Ae. aegypti* males	47.067	5.074	**<0.001**
Ae. aegypti females vs *An. gambiae* females	15.345	1.654	>0.05
***Ae. aegypti* females** vs *An. gambiae* males	53.464	5.196	**<0.001**
Ae. aegypti males vs ***An. gambiae* females**	62.412	6.399	**<0.001**
Ae. aegypti males vs *An. gambiae* males	6.397	0.597	>0.05
***An. gambiae* females** vs *An. gambiae* males	68.809	6.418	**<0.001**

References

1. Göpfert MC, Humphris ADL, Albert JT, Robert D, Hendrich O. Power gain exhibited by motile mechanosensory neurons in *Drosophila* ears. *Proc Natl Acad Sci U S A* **102**, 325-330 (2005).
